# Effects of Acute Cold Stress After Long-Term Cold Stimulation on Antioxidant Status, Heat Shock Proteins, Inflammation and Immune Cytokines in Broiler Heart

**DOI:** 10.3389/fphys.2018.01589

**Published:** 2018-11-13

**Authors:** Haidong Wei, Runxiang Zhang, Yingying Su, Yanju Bi, Xiang Li, Xin Zhang, Jianhong Li, Jun Bao

**Affiliations:** ^1^College of Life Science, Northeast Agricultural University, Harbin, China; ^2^College of Animal Science and Technology, Northeast Agricultural University, Harbin, China

**Keywords:** broiler heart, cold stimulation, antioxidant status, heat shock proteins, inflammatory factors, immune cytokines

## Abstract

To investigate the effects of acute cold stress (ACS) on chicken heart after cold stimulation, female broilers were raised in either normal (C) or gradually decreasing temperatures (CS I and CS II) for 34 days followed by a 24 h ACS at 7°C. Cardiac tissues were collected from the pre-ACS and ACS time points to analyze the histopathological changes, antioxidant status and the expression of heat shock proteins, inflammatory factors and immune-related cytokines. The CS II heart tissues showed shrunken cell membranes and nuclei, disordered or ruptured myocardial fibers, higher MDA content and upregulation in HSP27, HSP40, HSP70, NF-κB, COX-2, PTGEs, iNOS, TNF-α and IL-4 mRNAs, and in protein levels of HSP40, NF-κB and iNOS and reduction in CAT, GSH-px and SOD activity, as well as HSP90 and IFN-γ levels compared to the control tissues before ACS. In contrast, the HSPs were significantly increased, and the inflammatory and immune related factors were unaltered prior to the ACS in the CS I compared to the C group. Following ACS, MDA content was significantly increased and antioxidant activity was significantly decreased in the CS I and CS II groups compared to the C group. The levels of HSP27, HSP70, HSP90, inflammatory factors and IL-4 were significantly reduced and that of IFN-γ was significantly increased in CS I broiler hearts; the reverse trends were seen in CS II relative to CS I. Compared to the pre-ACS levels, that of HSP27, HSP40, HSP60, inflammatory factors and IL-4 were increased and IFN-γ was decreased in the C and CS II groups after ACS. Therefore, cold stimulation at drastically lower temperatures induced cardiac damage, which was further aggravated by ACS. In contrast, cold stimulation at only 3°C lower than normal temperature improved the adaptability of the broilers to ACS.

## Introduction

In the present world, stress costs the poultry industry in increased mortality and morbidity and in productivity losses (Shini et al., 2010). The stress of low temperature (cold stress, CS) to broiler chickens is very common in northern of China, and has caused decreased growth performance and increased production costs (Zhang et al., 2016). Stress is also a defensive response which helps the body adapt to changes in the external environment (Zulkifli et al., 1995). Housing broilers at their early age in moderate decreased environmental temperature has been reported to have a long-lasting benefit to improve the ability to acclimatize better with stressors in their later life (Shahir et al., 2012). However, if the stress is serious or continues for a long period, it will cause damage to animal bodies, disrupt the physiological homeostasis, and alter an animal’s immune response and behavior (Odihambo Mumma et al., 2006; Hu and Guo, 2008; Shini et al., 2008). Therefore, it is significant to production to gain further insight into the molecular mechanisms by which CS or cold stimulation affects chickens (Nguyen et al., 2015).

Cold stress influences many cellular processes that in turn lead to physiological and immunological responses (Hangalapura et al., 2003). Cells can develop efficient stress responses by activating protein control systems (i.e., gene transcription, protein expression, and enzyme activity) or proceed into cell-death signaling pathways to cope with the environment (Nguyen et al., 2015). The responses of CS are controlled by complex molecular regulatory systems that are still not completely known in broilers. In terms of enzyme activity, several studies have shown that CS inhibits antioxidant enzymes like total superoxide dismutase (T-SOD), catalase (CAT), and glutathione peroxidase (GSH-px) (Spasić et al., 1993; Selman et al., 2000). Kaushik and Kaur (2003) found a significant reduction in the activities of SOD, GSH-px, and CAT in the heart and liver of rats subjected to CS.

At the molecular level, a group of stress proteins called heat shock proteins (HSPs) are generally increased synthesis. HSPs are highly conserved molecular chaperones that regulate folding/unfolding of proteins, as well as the assembly/disassembly of protein complexes to maintain normal housekeeping functions (Bernabò et al., 2011). The expression levels of HSPs are normally low under physiological conditions, but they are rapidly synthesized in response to various stimuli like cold and heat stress, oxidative stress and heavy mental toxicity, and improve cellular stress tolerance, maintain normal metabolism and increase their viability (Yu et al., 2008; Zhao et al., 2013a, 2017; Flees et al., 2017; Nguyen et al., 2017). Heat stress (37 ± 1°C) elevates HSP70 protein and mRNA expression levels in the heart, liver and kidney of broilers (Yu and Bao, 2008). Zhao et al. (2014) reported that low temperature (12 ± 1°C) stress upregulated several HSP (HSP27, HSP40, HSP60, HSP70, HSP90) mRNAs in the immune organs of chickens. The same CS treatment also damaged the antioxidant defense system in chicken heart and increased the expression levels of HSPs (Zhao et al., 2013a).

In addition, emerging evidence indicates that CS can trigger the inflammatory response (Zhao et al., 2013b). Nuclear factor-kappa B (NF-κB), an inducible transcription factor mainly expressed in lymphocytes, is one of the crucial mediators of inflammation and activates pro-inflammatory factors like cyclooxygenase-2 (COX-2), tumor necrosis factor-alpha (TNF-α), prostaglandin E synthases (PTGEs), and inducible nitric oxide synthase (iNOS) (Ko et al., 2017; Mitchell and Carmody, 2018). Fu et al. (2013) found that CS (12 ± 1°C) increased the expression of NF-κB and TNF-α mRNAs in quail. Zhao et al. (2013a) similarly reported increased levels of NF-κB, TNF-α and PTGEs mRNAs in broiler hearts in response to CS (12 ± 1°C). In addition, cold stimulation at 14–15°C increased the expression level of iNOS mRNA in broiler lungs (Teshfam et al., 2006).

Low expression of interferon-γ (IFN-γ) over time has been shown to accelerate T lymphocytes to produce inflammatory factors, which could further exacerbate chronic inflammatory responses (Weng et al., 2017). Significant increase in interleukin-4 (IL-4) would repress release of IFN-γ (Crusz and Balkwill, 2015). Our previous study showed that long-term cold stimulation induced IL-4 and decreased IFN-γ levels in the ileum of broilers, resulting in a presumed immune imbalance (Su et al., 2018), This strongly suggests an interaction between immune cytokines and inflammation influenced by CS.

Therefore, CS can disrupt the oxidant/antioxidant balance and activate HSP expression, which trigger an inflammatory response. However, it is unclear whether long-term adaptation of broiler chicken to low-temperatures can alleviate the damage caused by acute cold stress (ACS). To this end, we observed histopathological changes, antioxidant status and changes in the expression of HSPs, inflammatory factors and immune cytokines in broiler hearts in response ACS following long-term (34 days) exposure to low temperatures.

## Materials and Methods

### Animals and Experimental Design

**FIGURE 1:**
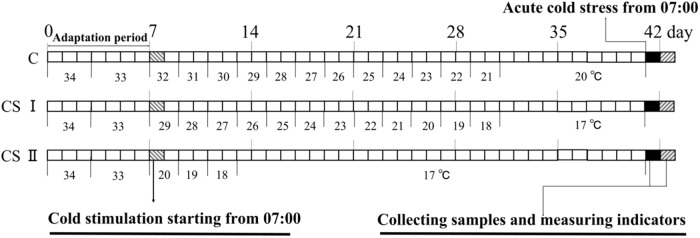
Cold stimulate procedure of three treatment groups.

Three-hundred and sixty 1-day-old AA female broilers were randomized into the control (C), cold stimulation I (CS I) and cold stimulation II (CS II) groups (*n* = 120, i.e., 4 × 30 per group). All birds were reared in battery cages in an environmentally controlled chamber. During the adaptive 1-week-long period, all groups were housed at the ambient temperatures of 34°C from day 1 to 3 and at 33°C from day 4 to 7. On the eighth day, the temperature for the C group was set to 32°C at 07:00 am and then gradually reduced by 1°C every 2 days till it dropped to 20°C on the 32nd day, and was maintained at that temperature till day 42. The CS I and II groups were, respectively, housed at 29°C and 20°C from the eighth day onward, and the temperatures were reduced by 1°C every 2 days to 17°C (reached on day 32 in CS I and day 14 in CS II), and thereafter maintained at that temperature till day 42. After 34 days (day 8 to 42) of cold stimulation (designated as pre-ACS), all broilers were exposed to ACS at 7°C for 24 h starting at 07:00 am on day 42. The temperature alteration scheme is shown in Figure 1. During the entire duration of the experiment, the broilers had free access to their feed and water, and the chamber humidity was set at 55–65%. Complete starter diet (CP: 21% and ME: 12.1 MJ/kg) was given in the first 3 weeks of age, and finishing diet (CP: 19.0% and ME: 12.6 MJ/kg) during the remaining 4–6 weeks. The light exposure duration was 23L:1D for the first 3 days and 16L:8D from the fourth day onward.

Two broilers from each replicate per group (*n* = 8) were randomly euthanized at 07:00 am on day 42 and 43, and their hearts were quickly removed, washed with deionized water and stored at -80°C till further use. All experiments were approved by and conducted according to the guidelines of the Institutional Animal Care and Use Committee of Northeast Agriculture University.

### Histopathologic Examination

#### Light Microscopic Examination

Heart tissues from the mid portion of the ventricular wall were fixed in 4% poly formaldehyde solution, dehydrated in ascending grades of alcohol, and then embedded in paraffin. Thin sections (5 μm) were prepared for staining with hematoxylin and eosin (HE) for observation. Histological samples were examined under a light microscope (XDS-1B, Olympus, Japan) and photos were taken under 200 times magnification.

#### Transmission Electron Microscope Examination

The cardiac tissues (about 1 mm × 1 mm × 1 mm) from the mid portion of the ventricular wall were rapidly fixed in 2.5% glutaraldehyde in PBS (v/v) for 3 h at 4°C, washed in 0.1 M sodium phosphate buffer (pH 7.2) for 1 h at 4°C, post-fixed in 1% osmium tetroxide (v/v) for 1 h at 4°C, and finally rinsed with the same buffer. The samples were dehydrated through ethanol and then acetone gradients, the latter with different proportions of pure acetone and epoxy resins, and then embedded in Epon. The embedded tissue samples were cut into ultra-thin sections (60–90 nm), stained with uranyl acetate and lead citrate, and then observed under transmission electron microscope (TEM, H-7650, Hitachi, Japan).

### Determination of Oxidative Stress Indices of Heart

The heart tissues (0.5 g pieces) from the mid portion of the ventricular wall at different time-points were homogenized in 4.5 mL saline (0.9% NaCl) and centrifuged at 700 ×*g* for 10 min. Supernatants were collected to determine antioxidant function. The activities of malondialdehyde (MDA), GSH-px, SOD, and CAT were measured in parallel with six replicates using the specific detection kits (Nanjing Jiancheng Technology Ltd., Nanjing, China) according to the manufacturer’s instructions. Absorbance of supernatants was detected at 532, 420, 550, and 405 nm, and results expressed as international units per mg of protein.

### RNA Extraction and Quantitative Real-Time PCR (qRT-PCR) Analysis

Total RNA was extracted from the heart tissues taken from the mid portion of the ventricular wall using RNAiso Plus Kit according to the manufacturer’s instructions (Takara, Dalian, China). The dried RNA pellets were re-suspended in 30 μL of 0.1% diethyl-pyrocarbonate (DEPC) treated water, and the concentration and purity of the total RNA were determined at 260 nm and 260/280 nm (Gene Quant 1300/100, United States), respectively. The first-strand complementary DNA (cDNA) was synthesized using oligo dT primer and Superscript II reverse transcriptase according to the manufacturer’s instructions (Takara, Dalian, China), and the cDNAs were diluted with sterile water and stored at -80°C till further use.

Primer Premier Software 5.0 (PREMIER Biosoft International, Palo Alto, CA, United States) was used to synthesize the specific primers for HSP90, HSP70, HSP60, HSP40, HSP27, COX-2, NF-κB, PTGEs, TNF-α, iNOS, IFN-γ, IL-4 and glyceraldehyde 3-phosphate dehydrogenase gene (GAPDH) genes. The primer sequences are shown in Table 1. The qRT-PCR was conducted using Light Cycler^®^ 96 qPCR system (Roche, Switzerland). Each 10 μL reaction mixture contained 5 μL 2X Roche Fast Universal SYBR Green Master kit (Roche, Switzerland), 1 μL diluted cDNA, 0.3 μL of each primer (10 μM) and 3.4 μL PCR grade water. The qPCR conditions were as follows: initial heating to 50°C for 2 min and 95°C for 10 min, followed by 40 cycles of 95°C for 15 s and 60°C for 1 min. The melting curve showed a single peak for each PCR product. The PCR reactions were performed in triplicate, and the threshold cycle (Ct) value used in subsequent calculations was the mean of the values from three reactions. The relative expression of each mRNA was calculated using the 2^-ΔΔCt^ method (Schmittgen and Livak, 2008), and the house-keeping gene GAPDH was used as the internal reference for normalization of the results.

**Table 1:** Gene-special primer sequences used for quantitative real-time PCR analysis.

Gene	Reference sequence	Primer sequences (5′–3′)
HSP27	NM_205290.1	Forward: ACACGAGGAGAAACAGGATGAG
		Reverse: ACTGGATGGCTGGCTTGG
HSP40	NM_001199325.1	Forward: GGGCATTCAACAGCATAGA
		Reverse: TTCACATCCCCAAGTTTAGG
HSP60	NM_001012916.1	Forward: AGCCAAAGGGCAGAAATG
		Reverse: TACAGCAACAACCTGAAGACC
HSP70	NM_001006685.1	Forward: CGGGCAAGTTTGACCTAA
		Reverse: TTGGCTCCCACCCTATCTCT
HSP90	NM_001109785.1	Forward: TCCTGTCCTGGCTTTAGTTT
		Reverse: AGGTGGCATCTCCTCGGT
TNF-α	NM_204267	Forward: GCCCTTCCTGTAACCAGATG
		Reverse: ACACGACAGCCAAGTCAACG
COX-2	NM_001167718	Forward: TGTCCTTTCACTGCTTTCCAT
		Reverse: TTCCATTGCTGTGTTTGAGGT
PTGEs	NM_001194983.1	Forward: GTTCCTGTCATTCGCCTTCTAC
		Reverse: CGCATCCTCTGGGTTAGCA
NF-κB	NM_205134	Forward: TCAACGCAGGACCTAAAGACAT
		Reverse: GCAGATAGCCAAGTTCAGGATG
IFN-γ	NM_205149.1	Forward: GCTGACGGTGGACCTATTATTGTAGAG
		Reverse: TTCTTCACGCCATCAGGAAGGTTG
iNOS	NM_204961.1	Forward: CCTGGAGGTCCTGGAAGAGT
		Reverse: CCTGGGTTTCAGAAGTGGC
IL-4	NM_001007079.1	Forward: GAGAGCCAGCACTGCCACAAG
		Reverse: GTAGGTCTGCTAGGAACTTCTCCATTG
GAPDH	NM_204305.1	Forward: GCACGCCATCACTATCTTCC
		Reverse: CATCCACCGTCTTCTGTGTG

### Western Blot Analysis

Frozen broiler heart tissues (100 mg) homogenized at low temperature and were then extracted using lysis buffer containing 1 mM PMSF (Beyotime Institute of Biotechnology, Shanghai, China). Homogenates were centrifuged at 12,000 ×*g* for 5 min to collect the supernatants. The supernatants were quantified with the Enhanced BCA Protein Assay Kit (Beyotime, China). For Western blotting, equal quantities of total protein (50 μg) were subjected to SDS-PAGE under reducing conditions on 12% gels. The separated proteins were transferred on nitrocellulose membranes using a DYCP-40C semi-dry transfer apparatus (LIUYI, Beijing, China). Membranes were blocked with 5% skim milk at 37°C for 2 h and then incubated overnight with primary antibodies against HSP40 (1:500, Abcam, United Kingdom), HSP60 (1:5000, kindly performed by Professor Shiwen Xu, Northeast Agricultural University, Harbin, China), and HSP70 (1:5000, kindly performed by Professor Shiwen Xu), NF-κB (1:200, Santa Cruz Biotechnology, United States), iNOS (1:500, Proteintech, United States) and GAPDH (1:5000, Bioss Antibodies, Beijing, China). The primary antibodies were subsequently localized with a horseradish peroxidase conjugated goat anti-rabbit IgG (1:10000, Bioss Antibodies, Beijing, China). The signal was measured by a chemiluminescence detection kit (Beyotime, China). The intensities of bands were quantified by a gray scale scanner (GeneGnome XRQ; Syngene Corp., Cambridge, United Kingdom) and the relative abundance of the proteins were expressed as the ratios of optical density of each of these proteins to that of GAPDH.

### Statistical Analysis

Statistical analyses were performed using SPSS 21 for Windows (SPSS Inc., Chicago, IL, United States). All data were tested for normal distribution using the Kolmogorov–Smirnov test. Inter-group and intra-group differences at the same and different time points, respectively, were analyzed by one-way ANOVA with Duncan’s multiple comparison. The results were expressed as mean ± SEM, and differences were considered statistically significant at *P* < 0.05.

## Results

### Histopathological and Ultrastructural Observation of Broiler Heart Tissue

**FIGURE 2:**
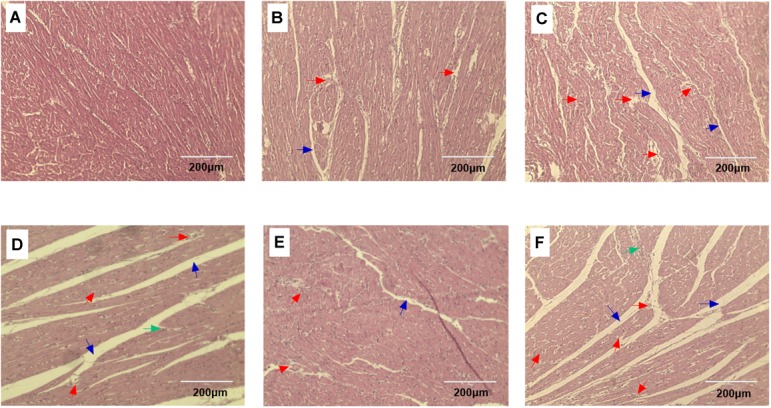
HE stained heart tissue sections of broiler after pre-ACS and ACS treatment. **(A–C)** Represent the C, CS I, and CS II groups at pre-ACS. **(D–F)** Represent the C, CS I, and CS II groups at ACS, respectively. (HE, ×200). Red arrows: disordered or ruptured cardiac muscle fiber arrangement. Green arrows: inflammatory cell infiltration. Black arrows: interstitial space.

Histopathology changes in the heart tissue samples following ACS compared to the pre-ACS state (Figure 2). The pre-ACS heart tissues suggested the regular and clear cardiac muscle fibers and myocardial structures in C group (Figure 2A). Heart tissue of CS I group at pre-ACS with slightly disordered myocardial fiber arrangement and interstitial space (Figure 2B), while that of CS II group showed clear lesions with disordered myocardial fiber arrangement, interstitial space and fragmentation (Figure 2C) at pre-ACS. The ACS heart tissues of C and CS II groups showed severe lesion and inflammatory cell infiltration (Figures 2D,F). The ACS histopathology observation of heart tissues in CS I group was similar to the pre-ACS, which exhibited slightly disordered or ruptured myocardial structures and cardiac muscle fibers (Figure 2E).

**FIGURE 3:**
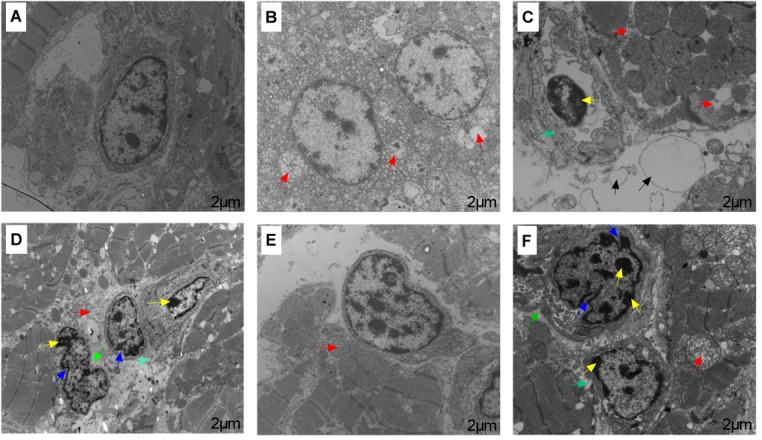
Transmission electron microscopy results of broiler heart tissues from C, CS I, and CS II groups after pre-ACS and ACS treatment. **(A–C)** Represent the C, CS I, and CS II groups at pre-ACS. **(D–F)** Represent the C, CS I, and CS II groups at ACS, respectively. Red arrows: slightly mitochondrial cristae disappearance, mitochondrial vacuoles and swelling. Yellow arrows: chromatin aggregation in the nuclear membrane and nuclear pore. Blue arrows: nuclear shrinkage. Green arrows: cytomembrane sinking. Black arrows: vacuolation of the cytoplasmic reticulum and edema. Magnification: 10,000×.

Transmission electron microscope showed ultrastructural changes in the heart tissue samples following ACS compared to the pre-ACS state (Figure 3). The pre-ACS hearts of the C group exhibited normal nuclei with uniform and finely dispersed chromatin, surrounded by cytoplasm containing clear and normal mitochondria (Figure 3A). The pre-ACS karyomorphology of CS I showed slightly swollen mitochondria and with disappearance of mitochondrial cristae (Figure 3B). CS II showed clear chromatin aggregation in the nuclei, vacuolation of the cytoplasmic reticulum and mitochondrial vacuoles and swelling (Figure 3C). Following ACS, severe shrinkage was seen in the nuclear and cytomembrane structures of the heart tissues of C group broilers (Figure 3D). While CS I heart (Figure 3E) showed similar features denoted in Figure 3B, the heart tissues of CS II showed nuclear shrinkage, partial loss of mitochondrial cristae and chromatin condensation (Figure 3F).

### Effects of ACS on Oxidative Stress Indices in the Heart Tissues

The changes in MDA content and activities of GSH-px, SOD, and CAT in broiler hearts are shown in Table 2. The pre-ACS and ACS heart MDA levels were significantly higher in the CS I and II groups compared to the C group (*P* < 0.05 for all), and the pre-ACS level in CS II was significantly higher compared to that in CS I (*P* < 0.05). Furthermore, the MDA content in each group increased post ACS compared to the pre-ACS levels (*P* < 0.05). Concomitant with the above results, the pre-ACS and ACS activities of GSH-px and SOD were significantly lower in the CS I and II groups compared to C, with the lowest activity seen in CS II (*P* < 0.05 for all). While CAT activity was similar between C and CS I (*P* > 0.05), that in CS II was significantly lower compared to both (*P* < 0.05). Antioxidant activity fluctuated in each group following ACS compared to pre-ACS levels, SOD and CAT activity significantly decreased in the C and CS II groups (*P* < 0.05) and showed a tendency to decrease in the CS I group. In contrast, GSH-px activity significantly increased in the C and CS II groups (*P* < 0.05) and significantly decreased in the CS I group (*P* < 0.05) after ACS compared to pre-ACS.

**Table 2:** Malondialdehyde content and activities of antioxidant enzymes in broiler hearts at pre-ACS and ACS.

	C	CS I	CS II	*P*-value
**MDA (nmol/mg prot)**				
pre-ACS	1.16^cy^± 0.10	2.02^by^± 0.05	2.30^ay^± 0.16	<0.001
ACS	2.33^bx^± 0.14	3.35^ax^± 0.06	3.49^ax^± 0.07	<0.001
*P*-value	<0.001	<0.001	<0.001	
**GSH-px (U/mg prot)**				
pre-ACS	265.22^ay^± 9.31	252.14^bx^± 8.02	154.54^cy^± 4.18	<0.001
ACS	325.32^ax^± 3.74	177.21^by^± 10.06	179.15^bx^± 5.27	<0.001
*P*-value	<0.001	<0.001	0.042	
**SOD (U/mg prot)**				
pre-ACS	91.84^ax^± 2.33	76.32^bx^± 1.72	58.91^cx^± 4.80	<0.001
ACS	60.91^ay^± 1.01	52.58^by^± 1.68	45.25^cy^± 2.34	<0.001
*P*-value	<0.001	<0.001	<0.001	
**CAT (U/mg prot)**				
pre-ACS	137.89^ax^± 4.09	113.74^a^± 2.80	86.96^bx^± 1.38	<0.001
ACS	112.76^ay^± 6.77	111.37^a^± 5.73	42.64^by^± 5.07	<0.001
*P*-value	<0.001	0.272	<0.001	

*^a,b,c^Means with different superscripts in a row within the treatment groups (C, CS I, and CS II) are significant differences (*P* < 0.05). ^*x,y*^Means with different superscripts in a column within the varieties (pre-ACS and ACS) are significant differences (*P* < 0.05), the same or no superscripts represent no significant differences (*P* > 0.05)*.

### Expression Levels of HSP in Broiler Hearts

**FIGURE 4:**
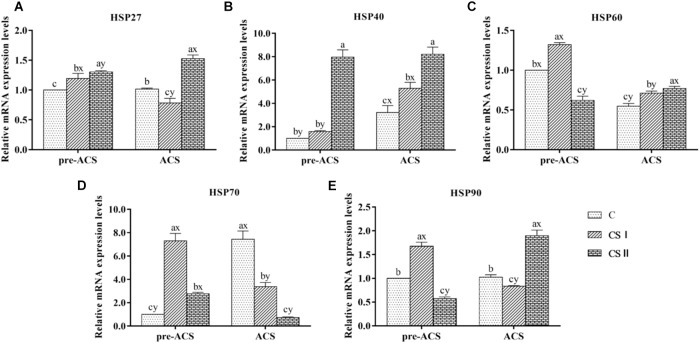
**(A–E)** Relative mRNA expression levels of HSP27, HSP40, HSP60, HSP70, and HSP90 in broiler hearts at pre-ACS and ACS. ^a,b,c^Means with different superscripts represent significant differences between groups at same time (*P* < 0.05). ^x,y^Means with different superscripts represent significant differences between pre-ACS and ACS in same group (*P* < 0.05), the same or no superscripts represent no significant differences (*P* > 0.05).

The pre-ACS and ACS levels of HSPs (including HSP27, HSP40, HSP60, HSP70, and HSP90) mRNAs in broiler hearts are shown in Figure 4. When compared with the C group, all HSPs were upregulated in the CS I and II groups (*P* < 0.05), except HSP60 and HSP90 that were downregulated in CS II (*P* < 0.05). HSP27 and HSP40 were most highly expressed in CS II, while HSP60, HSP70, and HSP90 were the highest in CS I at pre-ACS. Following ACS, HSP27, HSP40, HSP60, and HSP90 mRNA levels were higher and that of HSP70 was significantly lower in CS II compared to C group (*P* < 0.05 for all). However, the expression levels of HSPs in CS I were not consistent at ACS; while HSP27, HSP70, and HSP90 levels were lower compared to C group (*P* < 0.05), HSP40 and HSP60 levels were significantly higher (*P* < 0.05). Furthermore, the pre-ACS expression levels of all HSPs in CS I were lower compared to ACS values (*P* < 0.05). In contrast, pre-ACS and ACS HSP27, HSP40, HSP60, and HSP90 levels were higher (*P* < 0.05) and HSP70 was lower (*P* < 0.05) in CS II compared to the C and CS I groups.

**FIGURE 5:**
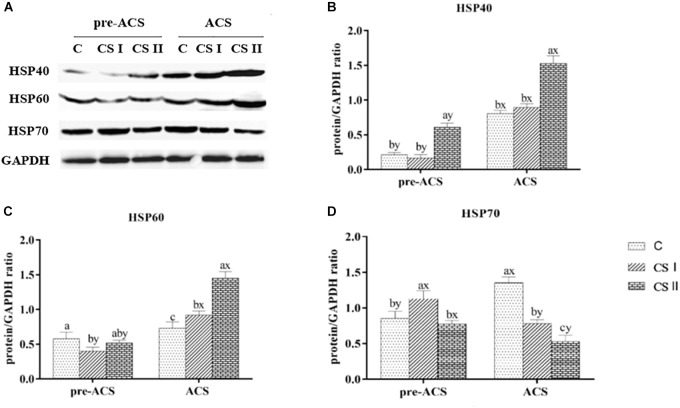
**(A)** Protein bands of HSPs and GAPDH. **(B–D)** Expression levels of HSP40, HSP60, and HSP70 proteins in broiler hearts at pre-ACS and ACS. ^a,b,c^Means with different superscripts represent significant differences between groups at same time (*P* < 0.05). ^x,y^Means with different superscripts represent significant differences between pre-ACS and ACS in same group (*P* < 0.05), the same or no superscripts represent no significant differences (*P* > 0.05).

The protein levels of HSP40, HSP60, and HSP70 in the broiler hearts at pre-ACS and ACS are shown in Figure 5. Similar to the expression levels of HSP40 and HSP70 mRNAs. The pre-ACS and ACS protein level of HSP40 was significantly increased (*P* < 0.05) in CS II group, while that in CS I group was no difference (*P* > 0.05), compared to C group. Compared with C group, the protein level of HSP60 was significantly decreased (*P* < 0.05), but that of HSP70 was significantly increased (*P* < 0.05) in CS I group at pre-ACS, while the pre-ACS protein levels of HSP60 and HSP70 in CS II group were similar (*P* > 0.05) to C group. The ACS expression of HSP60 in CS I and CS II groups was increased (*P* < 0.05) compared to C group. Conversely, the expression of HSP70 at ACS was decreased (*P* < 0.05) in CS I and CS II groups compared to C group. Compared to pre-ACS, the ACS protein levels of HSP40 and HSP60 were increased (*P* < 0.05) in each of group, while that of HSP70 was decreased (*P* < 0.05).

### Expression Levels of Inflammatory Factor in Broiler Hearts

**FIGURE 6:**
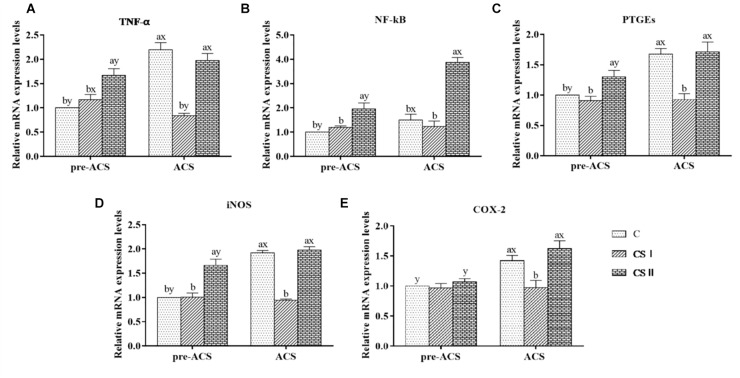
**(A–E)** Relative mRNA expression levels of TNF-α, NF-κB, PTGEs, iNOS, and COX-2 in broiler hearts at pre-ACS and ACS. ^a,b,c^Means with different superscripts represent significant differences between groups at same time (*P* < 0.05). ^x,y^Means with different superscripts represent significant differences between pre-ACS and ACS in same group (*P* < 0.05), the same or no superscripts represent no significant differences (*P* > 0.05).

The pre-ACS and ACS levels of TNF-α, NF-κB, PTGEs, iNOS, and COX-2 mRNA in the broiler hearts are shown in Figure 6. The pre-ACS relative levels of the pro-inflammatory factor mRNAs in the CS I group were similar (*P* > 0.05), and that in the CS II group were markedly increased (*P* < 0.05) excepted COX-2 with no difference (*P* > 0.05) compared to group C. Following ACS, the expression levels of TNF-α, PTGEs, iNOS, and COX-2 were significantly higher in the C and CS II groups compared to the CS I group (*P* < 0.05), and similar between C and CS II (*P* > 0.05). The expression of NF-κB was no difference in C group (*P* > 0.05), and that in CS II group was increased (*P* < 0.05) compared with CS I group. Compared to the pre-ACS levels, the inflammatory factors were significantly upregulated in the C and CS II groups after ACS (*P* < 0.05), and largely unaltered in the CS I group (*P* > 0.05).

**FIGURE 7:**
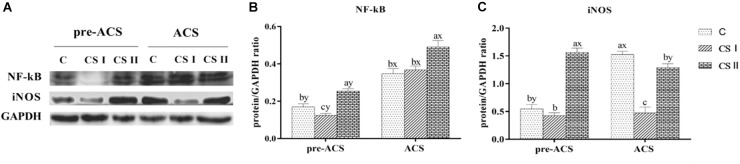
**(A)** Protein bands of NF-κB, iNOS, and GAPDH. **(B,C)** Expression levels of NF-κB and iNOS proteins in broiler hearts at pre-ACS and ACS. ^a,b,c^Means with different superscripts represent significant differences between groups at same time (*P* < 0.05). ^x,y^Means with different superscripts represent significant differences between pre-ACS and ACS in same group (*P* < 0.05), the same or no superscripts represent no significant differences (*P* > 0.05).

Protein levels of NF-κB and iNOS in the broiler hearts at pre-ACS and ACS are shown in Figure 7. Similar to the mRNA results, expression of NF-κB at pre-ACS, and iNOS at pre-ACS and ACS were significantly decreased (*P* < 0.05) in CS I group. Conversely, expression of NF-κB at pre-ACS and ACS, and iNOS at pre-ACS were significantly increased (*P* < 0.05) in CS II group. Compared to pre-ACS, the protein level of NF-κB in ACS was increased in each group and expression of iNOS in ACS C group was increased (*P* < 0.05). No difference was observed in CS I group (*P* > 0.05), and expression in the CS II group was decreased (*P* < 0.05) compared to pre-ACS.

### Expression Levels of IL-4 and IFN-γ in Broiler Hearts

**FIGURE 8:**
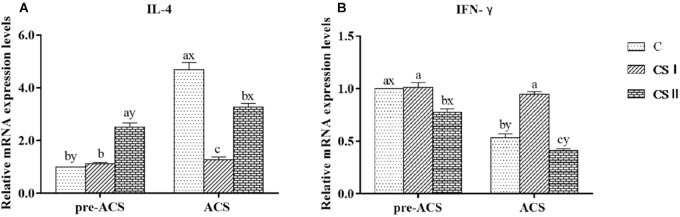
**(A,B)** Relative mRNA expression levels of IL-4 and IFN-γ in broiler hearts at pre-ACS and ACS. ^a,b,c^Means with different superscripts represent significant differences between groups at same time (*P* < 0.05). ^x,y^Means with different superscripts represent significant differences between pre-ACS and ACS in same group (*P* < 0.05), the same or no superscripts represent no significant differences (*P* > 0.05).

The relative expression levels of IL-4 and IFN-γ mRNAs were analyzed to determine the immune responses of pre-ACS and ACS in broiler hearts (Figure 8). The pre-ACS levels of IL-4 and IFN-γ were similar between the C and CS I groups (*P* > 0.05), whereas that of IL-4 and IFN-γ were, respectively, increased and decreased in CS II compared to the other two groups (*P* < 0.05 for all). After ACS, the relative expression of IL-4 was significantly lower, and that of IFN-γ was significantly higher in CS I compared to C and CS II groups (*P* < 0.05 for all). Compared to the pre-ACS levels, IL-4 was upregulated (*P* < 0.05) and IFN-γ was downregulated (*P* < 0.05) after ACS in the C and CS II groups. In contrast, there was no difference in the pre- and post-ACS levels of the cytokine mRNAs in the CS 1 group (*P* > 0.05).

## Discussion

Currently, it has been reported that CS may cause an estimated total annual economic loss to the Chinese poultry industry of 100 million (Chen et al., 2014). In an attempt to better understand the mechanisms underlying the adaptation and acclimatization to long-term cold stimulus and followed ACS, we measured histopathological changes, antioxidant status and gene levels of HSPs, inflammatory factors and immune cytokines in hearts of three broiler groups.

Exposure to CS, especially in early life, causes cell and tissue damage in chicken broilers (Julian, 2000). Studies also show that CS at 12°C causes histological damage of duodenum (Zhang et al., 2011) and intestinum tenue (Zhao et al., 2013b) in broilers. In our study, we exposed 1-day-old broilers to 34-day cold stimulation followed by a 24 h ACS at 7°C. Histopathological assessment indicated severe organelle distortion and shrinkage, disordered or ruptured cardiac muscle fibers, and interstitial space in the C group after ACS; the sudden drop in temperature from an ambient 20°C likely caused the significant cellular damage. Serious alterations were also in the nuclear, mitochondrial and cardiac muscle fibers structure of CS II heart tissues, indicating that the prolonged exposure to 17°C resulted in a chronic CS state that caused the ultrastructural changes. In contrast, no significant pathomorphological changes were seen in the CS I group since the broilers were able to adapt to the gradually decreasing temperatures over a longer period of time, and the sudden drop to 7°C did not lead to severe ACS.

Stressful environmental conditions can increase the level of reactive oxygen species (ROS) in animal tissues (Yang et al., 2013), and the excessive production of ROS overwhelms the antioxidant system, which lead to oxidation of unsaturated fatty acids and generates MDA (Lykkesfeldt and Svendsen, 2007). SOD and CAT are important cellular antioxidants that protect cells from oxidative stress damage (Hermes-Lima et al., 1998), wherein SOD reduces the superoxide radicals into hydrogen peroxide, which is then decomposed into water and oxygen by CAT. Jia et al. (2009) reported that CS at 12°C disrupted the oxidant/antioxidant balance and resulted in MDA content increase, as well as SOD and GSH-px activity in chicken lungs. In our study, MDA levels increased significantly both during pre-ACS and after ACS, which is consistent with the findings of Jia et al. (2009), indicating that cold stimulation caused oxidative stress in broiler hearts. In contrast to the results of Jia et al. (2009) and Li et al. (2017), however, the activities of all antioxidant enzymes were decreased in the CS I and CS II groups at both the pre-ACS and ACS time points. In the CS II group especially, the antioxidant activity was significantly decreased compared to that in CS I. A likely explanation for this is the fact that the CS II group broilers were exposed to a more drastic cold stimulation which further inhibited the antioxidant system (Spasić et al., 1993; Selman et al., 2000). Ramnath and Rekha (2009) found that chickens exposed to the low temperature of 4 ± 1°C 6 h per day for 5–10 days had significantly reduced SOD and GSH-px activity. Furthermore, Pajovič et al. (2006) found that the CS of 4°C for 2 h also decreased SOD activity. These results indicate that cold stimulation increase the production of oxidizing substances which cannot be completely eliminated by the antioxidant system, leading to further inhibition of the antioxidant enzymes. Although the 24 h ACS in our study inhibited the antioxidant enzymes in the CS I and CS II, it was slight in the CS I broilers indicating milder CS and stronger antioxidant function against this stress compared to CS II.

Yang et al. (2016) and Zhao et al. (2016) found that oxidative stress was often associated with high levels of HSPs including HSP27, HSP40, HSP60, HSP70, and HSP90. It was also reported that CS-induced oxidative stress further increased the expression of HSPs and triggered the inflammatory response (Fu et al., 2013; Zhao et al., 2013a). HSP70 is the most abundant HSP, and mediates the folding, assembly and modification of newly synthesized proteins under stressful conditions (Walsh et al., 2004). HSP40 acts as a molecular chaperone and regulates the activity of HSP70 ATP enzyme (Hennessy et al., 2005; Qiu et al., 2006), while HSP90 maintains the configuration of intracellular proteins (Chen and Meyrick, 2004; Whitesell and Lindquist, 2004). HSP60 exists in a stable state in the cytoplasm and mitochondrial matrix under normal conditions, and rapidly activates to repair the denatured proteins under stress (Itoh et al., 2002). The major function of HSP27 is the repair of cardiac damage (Michaud et al., 1997). Therefore, the damage and repair processes in various organs are often accompanied by upregulation of HSPs (Khoso et al., 2015; Liu et al., 2015; Yang et al., 2016). We found that the expression levels of most HSPs were higher in the CS I and CS II groups compared to the C group after the 34-day cold stimulation, with highest levels of HSP27 and HSP40 in CS II, and that of HSP60, HSP70, and HSP90 in the CS I group. Long-term cold exposure would likely upregulate the macromolecular HSPs (HSP60, HSP70, and HSP90) in order to maintain various cellular functions and help the cells adapt to the new environment. However, the lower expression levels of HSP70 and HSP90 in the CS II compared to the C group indicates that violent cold stimulation for a long time may induce dysfunctional HSPs, which then lose their ability of anti-stress adaptation. In addition, high expression of the small molecule HSP27 and HSP40 may inhibit the expression of macromolecular HSPs. After the 24 h ACS, HSP70 was downregulated, and HSP27, HSP40, HSP60, and HSP90 were upregulated in the CS II broilers, indicating a serious cellular damage caused by ACS. Taken together, cold stimulation at much lower than ambient temperatures caused serious stress or damage to the broiler hearts, and altered the expression of the HSPs. We hypothesize that after 34 days of cold stimulation, the CS I broilers may have adapted to the cold, which protected them against ACS and obviated the need for elevated HSP levels after ACS. In contrast, the CS II broilers had not adapted to the colder temperatures, which disrupted HSP expression.

The inflammatory response is a complex process involving a number of signaling pathways, of which the NF-κB pathway is the master regulator (Zhang et al., 2014). The NF-κB is a transcription factor which controls inflammatory and immune responses by regulating target genes such as COX-2, iNOS, and TNF-α (Ko et al., 2017; Mitchell and Carmody, 2018). TNF-α plays a central role in inflammation, immunity and apoptosis, and is released upon stress-related stimuli (Bayrakli et al., 2012). COX-2 is induced by various stimuli and triggers the production of a number of pro-inflammatory prostaglandins at the site of inflammation (Tsatsanis et al., 2006). Prostaglandins are responsible for the typical inflammatory symptoms like pain, redness, and swelling (Aoki and Narumiya, 2012). Fu et al. (2013) reported that chronic and ACS increased the expression levels of NF-κB and TNF-α mRNA in the intestinal tract of quail and induced an inflammatory response. Furthermore, CS also led to intestinal inflammation in rats (Kaushik and Kaur, 2005). We found that NF-κB, PTGEs, iNOS, COX-2, and TNF-α mRNAs were significantly upregulated after the 34-day cold stimulation and after the 24 h ACS in the CS II, but there were no marked differences in CS I. This indicated that cold stimulation induced relatively serious stress in the hearts of the CS II broilers which led to the inflammatory response. In contrast, the cold stimulation conditions were relatively mild for the CS I broilers, which enabled them to gradually adapt to the low temperatures and therefore did not affect the inflammatory factors.

IFN-γ and IL-4 are the typical cytokines secreted by Th lymphocyte subclasses Th1 and Th2 cells and other immune cells, respectively, and the abnormal production of either affects immune and inflammatory responses (Sofi et al., 2009). CS of 12 ± 1°C for 20 days upregulated IL-4 and downregulated IFN-γ mRNAs in the ileum and duodenum of broilers (Zhao et al., 2013b). Our previous study showed that cold stimulation of broilers at 12°C lower than the ambient temperature triggered an inflammatory response and caused the reduction in IFN-γ and elevation in IL-4 levels (Su et al., 2018). Consistent with these findings, the current study showed that the expression of IL-4 mRNA was significantly increased and that of IFN-γ mRNA was clearly decreased in the CS II broiler hearts, while no difference was seen in CS I, either after the 34-day cold stimulation or 24 h ACS. This indicated that cold stimulation temperatures much lower than the ambient significantly caused stress and induced a shift in the cytokines balance in the heart of broilers, while the cold stimulation at only 3°C lower than the ambient did not cause any changes. It is believed that severe CS can alter production cytokines lead to dysregulation of cell-mediated immune responses. Thus, CS affects-modulates the immune system (i.e., it suppresses immune function under some conditions while enhancing it under others) may be associated with stress-induced cytokine changes depending on the type, degree, and duration of stress.

## Conclusion

Cold stimulation at 12°C lower than the ambient temperature is stressful for broilers, resulting in long-term oxidative stress, ultrastructural cardiac damage, immune dysregulation and inflammation, which are aggravated by ACS. In contrast, cold stimulation at only 3°C lower than the ambient temperature increased the adaptability of the broilers to the subsequent ACS, by relieving the oxidative stress, reducing the levels of HSPs and pro-inflammatory factors, and maintaining the immune balance.

## Author Contributions

HW, RZ, and JB designed the experiments. YS and YB performed the molecular studies. HW and XZ bred and managed the experimental animals. HW and YB analyzed the data. HW, RZ, and JL wrote the first draft of the manuscript in conjunction with other authors. XL and JL supervised the process of this work. All authors read and approved the final manuscript.

## Conflict of Interest Statement

The authors declare that the research was conducted in the absence of any commercial or financial relationships that could be construed as a potential conflict of interest.
